# Mindful energy and information flow: A reflective account of S.E.L.F connection during COVID-19

**DOI:** 10.1177/1473325020973302

**Published:** 2021-03

**Authors:** James J Lucas

**Affiliations:** School of Health and Social Development, Deakin University, Geelong, Australia

**Keywords:** Mental health, emotions, loss, wellbeing, social work education, reflective practice

## Abstract

Life during the COVID-19 pandemic is uncertain, intense, and traumatic. At the same time, there is room for hope, inspiration, and meaning for social workers through mindfully connecting with energy-information flow as it influences our Safety, Emotions, Loss, and Future – S.E.L.F. As adapted from the Sanctuary Model®, this S.E.L.F connection is an opportunity to discover within ourselves our unwavering core that is grounded, present, and connected and sustain an ethical and compassionate approach to social work practice, education, and research during this time of pandemic. The aim in this reflective essay is to provide an example of S.E.L.F. connection from the perspective of a Buddhist and social work academic at an Australian university during the COVID-19 pandemic. While beneficial, ongoing S.E.L.F. connections are necessary for social workers if we are to stay mindful of energy-information flow and steer this flow towards the creation of a story of relationship, compassion, and connection into the future.

A connection with one’s self is critical for social workers who, in their roles as practitioners, educators, and researchers, expose themselves to situations of vulnerability, uncertainty, and trauma in the pursuit of social justice. This connection however, requires being mindful of the energy and information flow within and between the mind, body, and relationships (Baldini et al., 2014; McGarrigle and Walsh, 2011; Shier and Graham, 2010) as it influences experiences of Safety, Emotions, Loss, and Future (i.e., S.E.L.F) (Bloom and Sreedhar, 2008; Esaki et al., 2013). With the advent of the COVID-19 pandemic, a mindful stance towards S.E.L.F is of upmost importance if social workers are to maintain ethical practice, promote resilience, and avoid burnout. The aim in this paper is to provide a reflexive account of S.E.L.F connection as a Buddhist and social work academic at an Australian university during the COVID-19 pandemic.

## Energy-information flow: Mind, body, relationships

Siegel (2015) argued, in his Interpersonal Neurobiology (IPNB) framework, that we are part of a dynamic system of energy-information that constitutes and “flows” through our minds, embodied brain, and relationships. From an IPNB perspective, energy is the capacity to do something, while information is the form that energy takes on, or as Siegel (2019) termed, energy-in-formation. The mind is an embodied and emergent process in that it is distributed through the brain and broader nervous system (embodied), consists of a flow of energy and information (process), but cannot itself be found within but nonetheless its existence is dependent on the body and the energy-information flow (emergent). Relationships are the foundation for us to share energy and information from one mind to another in the form of our communication (verbal and non-verbal) and behaviour and is therefore dependent, again, on the brain/body as the physical mechanism for that sharing (Siegel, 2015).

The state of energy-information flow between these three factors: (1) the mind, (2) the brain/body, and (3) our relationships is indicative of a person’s state of wellbeing. In strengthening wellbeing, Siegel (2015) stated that through the human mind’s ability to see itself, or “mindsight”, as a flow of energy-information we can both monitor and regulate that flow as necessary. Mindsight is associated with a present-moment groundedness and a mental defusion between the person as the observer and their mind-body-relationships as the observed that enables a person to exert a great deal of control or regulation over habitual ways of thinking, feeling, and acting. The ability however, to maintain mindsight on the state of energy-information flow within and between one’s mind, body, and relationships is dependent on the conscious application of mindfulness (Black, 2011; Siegel, 2009). This in turn, facilitating an increased awareness and regulation over energy-information flow in a manner that creates space for the alignment of actions with their life values (Baldini et al., 2014; Purser and Milillo, 2015; Siegel et al., 2011; Thompson et al., 2011).

## Mindfulness

Lucas (2017: 276) defined mindfulness as “remembering to pay attention to something in one’s awareness”. It is important to note that mindfulness is not a product of western psychology, but rather has origins in Buddhist spiritual traditions where it is cultivated for the attainment of Enlightenment or Buddhahood – the state of compassionate omniscience in which reality is directly perceived as it truly exists: empty of inherent existence (Flanagan, 2011; Tenzin, 2014). While often referred to in the literature as a noun, researchers such as Grossman and van Dam (2011) consider its adjectival form (i.e., [being] mindful) more appropriate given its state-like, fluctuating nature. A person’s ability to be mindful supports their attainment of Enlightenment or Buddhahood through holding their mindsight on the Dharma (i.e., the Gautama Buddha’s teachings) so much so that one’s mind “mixes” with the Dharma and is more fully integrated into everyday life (Tenzin, 2014).

In Buddhist spiritual traditions, the principal method to strengthen mindfulness is through meditation (Kornfield, 2008), of which there are generally two types: (1) Shamatha, and (2) Vipassana (Tenzin, 2014). Shamatha mediation is a form of attention regulation training in which the meditator focuses on a chosen object single-pointedly such as their body, breath, or thoughts and emotions. In order to continuously focus single-pointedly, the meditator requires strong mindfulness to anchor their attention on the chosen object, without which their mind becomes distracted and the mediation object lost. In Vipassana meditation, the single-pointed concentration developed in Shamatha is directed at a new object: the true nature of reality being both interdependent and insubstantial, in order to attain the ultimate goal of Enlightenment. Without training one’s mindfulness however, Tenzin (2014) argued that people’s happiness will remain dependent on external conditions such that they will continue to chase objects (physical and mental) perceived as pleasurable, avoiding those perceived as painful, and feeling coldly indifferent to everything else.

As COVID-19 pandemic has evolved, I as a Buddhist and social work academic have found benefit in strengthening and applying mindfulness to the flow of energy-information within and between my mind, body, relationships; particularly in terms of my sense of S.E.L.F or Safety, Emotions, Loss, and Future (Bloom and Sreedhar, 2008; Esaki et al., 2013).

## S.E.L.F connection and mindful energy-information flow

In a time of widespread suffering, fear, and uncertainty directly resulting from the COVID-19 pandemic, energy-information flow vacillates constantly between a state of chaos and rigidity. The only certainty at this time is that everything is uncertain. It is therefore a time that demands from me as a social work academic to maintain a mindful stance towards my S.E.L.F in sustaining a state of health and wellbeing and to support others in the same way. A key reflective exercise I utilised with students and myself is to explore our experiences of Safety (e.g., physical, mental, social, cultural, spiritual), Emotions (e.g., stress, anxiety, excitement, and hope), Loss (e.g., grief and preparing for change), and Future (e.g., personal narrative, values, meaning, and purpose) – “S.E.L.F” – as social workers during COVID-19, as adapted from the Sanctuary Model® (Bloom and Sreedhar, 2008; Esaki et al., 2013).

In class, I guide students (and indirectly myself) to reflect individually and in small groups over what safety, emotions, loss, and future means for them as emerging social workers, how these S.E.L.F aspects are affected by the COVID-19 pandemic, and how they can be nurtured moving forward. For example, how do you know you are safe and secure? How do you know when you are not safe and secure? (Safety); What does stress feel like for you? In what part/s of your body? How can you manage strong negative/positive emotions without placing your safety at risk? (Emotions); How can you prepare for change? How can you acknowledge grief? What does loss/grief feel like for you? (Loss); and what story are you trying to create for yourself? What values are you trying to embody as an emerging social worker? Why is studying social work important for you? Where do you want to head in the future? (Future).

The purpose of this exercise is to focus students’ mindsight, with use of their mindfulness, towards the state of energy-information flow within their mind, body, and relationships in order to cultivate their S.E.L.F. connection while studying and preparing for placement during the pandemic. A short excerpt of my own reflections of S.E.L.F connection in the context of my role as a social work academic during the COVID-19 pandemic is provided as an example.

### Safety

Most notably is the chaotic energy-information flow related to the risks that COVID-19 poses to my physical health and the health of my husband, family, and friends. The worry and anguish over this risk, together with the social isolation of lockdown and the impending economic crises, compounds the negative impact on my physical and mental health. As a Buddhist, the pandemic has forced to the centre of my awareness the four inherent sufferings of life: (1) birth, (2) aging, (3) sickness, and (4) death, which inspired the Buddha Gautama to seek permanent cessation of these sufferings and attain Enlightenment (Tenzin, 2014). Where usually my daily life soaked in the privilege of being a white, professional middle-class, able-bodied, gay male allows such experiences to be taken for granted or frankly ignored altogether, the COVID-19 pandemic has situated them at the forefront of my individual and our collective consciousness.

A call for being mindful of the fragility, preciousness, and impermanence of life is, as a result, made paramount as I consider my sense of safety. In viewing myself within a dynamic system of energy-information that flows within and between my mind, body, and relationships (Siegel, 2019), life for me extends beyond the physical form, to include elements such as my relationships with others, my spirituality, and the environment. As the pandemic accelerates globally, and locally we arrive in our “second-wave”, I experience turbulent energy-information flow on all fronts of my life, which emerges as a decreased sense of safety. In an attempt to maintain a mindful, “grounded” stance in such turbulence, and therefore increase a sense of safety, I view my experiences as an opportunity to practice Dharma (the Buddha’s teachings). In this way, a sense of meaning and purpose emerges in my daily life and I am able to keep putting one foot in front of the other each day.

### Emotions

The emotional form of energy-information flow is probably the most dynamic. The mental and emotional overload experienced from mass transitioning of learning and teaching to a fully-online mode while also maintaining current teaching, research, and service workload expectations; in particular, managing an emergency response in sustaining the social work field education placement program during the pandemic, has been extraordinary. Anxiety and anguish over ensuring current student placements can continue albeit in a remotely-supervised fashion (Crisp and Hosken, 2016) and planning for upcoming placements in an unpredictable environment has been a constant companion and kept me awake many a night. Despite this, hope for relief and success endures and emotional and practical support from my academic colleagues, husband, family, and friends has been unwavering. In experiencing this emotional storm, it has been an ongoing exercise in acceptance, compassion, and perseverance; particularly as our second-wave of the pandemic emerges locally.

On acceptance, I refer not to an “agreeance” of the situation in all its relentlessness, but rather a clear acknowledgement of its existence, emergence, and “flow” without resistance. The sickness, death, and upending of our lives that has arisen from the COVID-19 pandemic exists whether I agree with it or not. If therefore, I wish to maintain a sense of groundedness in my work and life amid the strong negative emotional experiences such as helplessness, anxiety, and depression, then I first need to mentally “hold” the reality of the pandemic with courage and acceptance. Through such a mindful acceptance of how the pandemic-related energy-information if flowing (whether chaotically, rigidly, or flexibly) within and between my mind, body, and relationships, I can better see what action, if any, I need to take.

#### Loss

The way in which I have experienced “loss” in the evolution of the COVID-19 pandemic is in a sense of “lack” of “absence” in certain aspects of the energy-information flow. What was or was possible is no longer or has changed. The full range of loss however, as Weenolsen (1988) argued, continues to be experienced on various layers ranging from the concrete physical loss (e.g., physical absence of social connection with work colleagues), to more relational and abstract losses (e.g., loss of a sense of control and predictability, loss of community connection). There has also been imposed loss (e.g., on-campus learning connections with students), ambiguous loss (e.g., unclear information and timing surrounding the pandemic), and anticipated loss (e.g., of my potential death and that of family and friends), all of which involve grief of varying intensities. In mindfully directing my mindsight towards the energy-information flow of these personal loss and grief experiences, the image of a lake emerges.

The water of this lake – my mind – interacts with the external world via the storms that distort its surface, the animals that drink from it, the rain that replenishes it, and the sun that warms it. Some interactions may even come from within the lake itself. The lake’s water is rarely still with each external world interaction creating ripples, waves, or perhaps freezing the water altogether, which in turn, interact with each other resulting in their amplification or cancellation. Resistance is futile and acceptance is essential. Acceptance here, again, is not an agreeance nor a passive resignation, but rather a strong and courageous facing of what is in order to know clearly and act meaningfully. What this experience has reinforced for me as a Buddhist and social work academic during the COVID-19 pandemic is the transiency or impermanence of all phenomena, including the phenomena that is my life, and the attachment held (i.e., grasping or resistance) to these thus resulting in the loss and grief that emerges and the future I am constructing for myself.

#### Future

In the face of the present uncertainty, stressors, and suffering there is still hope. There is still hope in the story I write for myself, choice in the values I hold, and opportunity in the meaning I create (Neimeyer et al., 2014). I ask myself, what am I trying to embody? What am I modelling to students? Where am I heading? In the wake of the COVID-19 pandemic and now in this local “second-wave”, what I have discovered is how this global, existential crisis has created an unending turbulence (and at times, a “freezing over”) of the energy-information flow constituting my sense of safety, emotions, and loss. I have also discovered how this crisis has forced in myself a reconnection with what resonate with my “core” – the still, grounded, compassionate core of my being.

What is uncovered from this mindful exploration is the realisation that what matters most to me is relationship, what matters is compassion, what matters is connection. I find these qualities indispensable in society and is what we as social workers defend and strive to promote in our practice, education, and research. Relationships, compassion, and connection is the story I seek to create, embody, and bring into this world. Relationships, compassion, and connection are my future.

## Epilogue: Ongoing S.E.L.F connections

Life during the COVID-19 pandemic is uncertain, intense, and traumatic. At the same time, there is room for hope, inspiration, and meaning. S.E.L.F connection (Bloom and Sreedhar, 2008; Esaki et al., 2013), as shown in this reflexive essay, being a process of mindfully connecting with energy-information flow within and between mind, body, and relationships (Siegel, 2019), is one possible path forward. It is an opportunity to discover within ourselves our unwavering core that is grounded, present, and connected. Ongoing S.E.L.F connections however, are necessary for social workers in practice, education, and research if we are to stay mindful of energy-information flow and steer this flow towards the creation of a story of relationship, compassion, and connection into the future.

## Data statement

There is no data associated with this project.
